# The Dental and Oral Significance of Hutchinson-Gilford Prfogeria Syndrome -

**DOI:** 10.31661/gmj.vi.3763

**Published:** 2025-01-20

**Authors:** Safa Saeed, Jawaher Abdulaziz Alhababi, Fatimah Alanazi, Amal Albarrak, Halh Alabdulmonem, Alanoud Inad Alenazi, Maha Alsane, Myle Akshay Kiran

**Affiliations:** ^1^ College of Dentistry, King Saud bin Abdulaziz University for Health Sciences, Riyadh 14611, Saudi Arabia; ^2^ Graduate, Doctor of Science in Dentistry Oral Medicine Track; ^3^ Department of Scientific Clinical Research, General and Alternative Medicine, Hospital and Health Care Administration

**Keywords:** Hutchinson-Gilford Progeria Syndrome, Lamin A, Oral Manifestations

## Abstract

**Background:**

Hutchinson-Gilford Progeria Syndrome (HGPS) is a rare genetic disorder caused by a point mutation in the LMNA gene that encodes lamin A. This mutation results in the production of progerin, a defective protein that accelerates cellular aging. This review explores the oral and dental manifestations of HGPS, emphasizing their role in early diagnosis, management strategies, and the potential for targeted therapies.

**Materials and Methods:**

A thorough review of existing literature was conducted to summarize the phenotypic characteristics, oral health implications, and current approaches for the management of HGPS-related complications.

**Results:**

Individuals with HGPS exhibit age-associated complications starting in early childhood, including distinct craniofacial abnormalities and severe oral health challenges. Common oral manifestations include delayed tooth eruption, microdontia, malocclusion, dental caries, tooth loss, and mandibular osteolysis. These abnormalities necessitate a multidisciplinary approach involving pediatric dentists, orthodontists, oral surgeons, and geneticists to deliver comprehensive care.

**Conclusion:**

Understanding the pathophysiological mechanisms behind HGPS oral anomalies is vital for improving diagnosis and treatment. Advances in genetic research hold promise for developing targeted interventions to alleviate dental complications and enhance the quality of life for affected individuals. Ongoing research and a collaborative care approach are essential to address the challenges posed by HGPS effectively.

## Introduction

**Figure-1:**
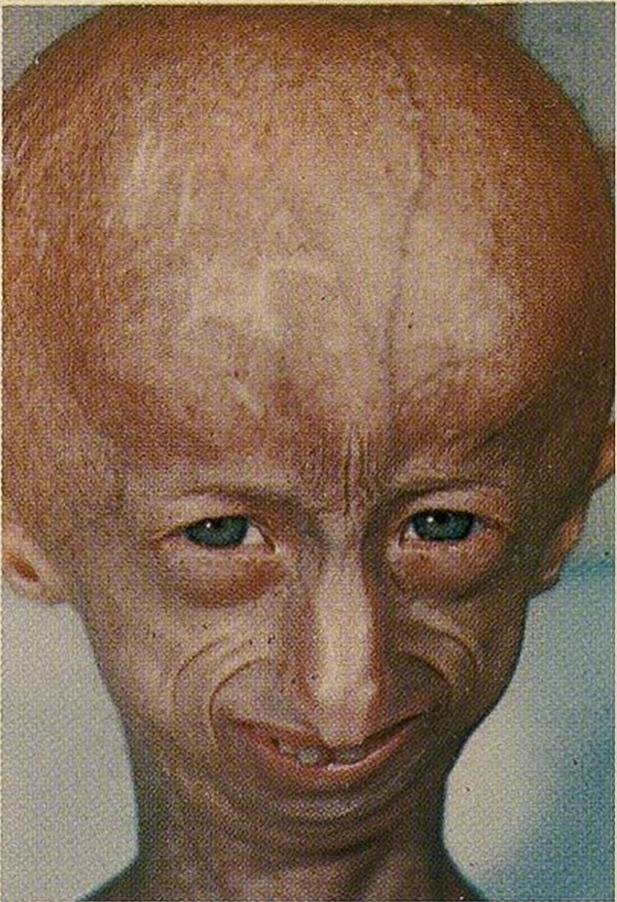
Figure 1. Disproportionate small face relative to the head. The skin appears wrinkled and thin.

**Figure-2:**
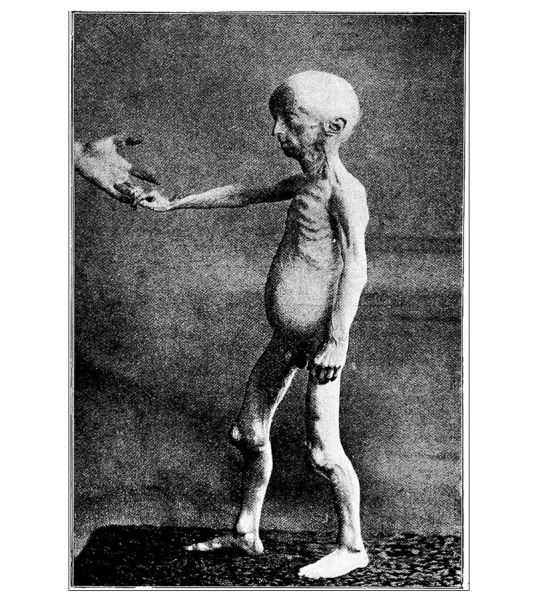
Figure 2. Notice the extreme emaciation and the conspicuous ribs, veins, and tendons. The abdomen is full, and the patellae are pushed forward by the relatively big lower ends of the femora.

**Figure-3:**
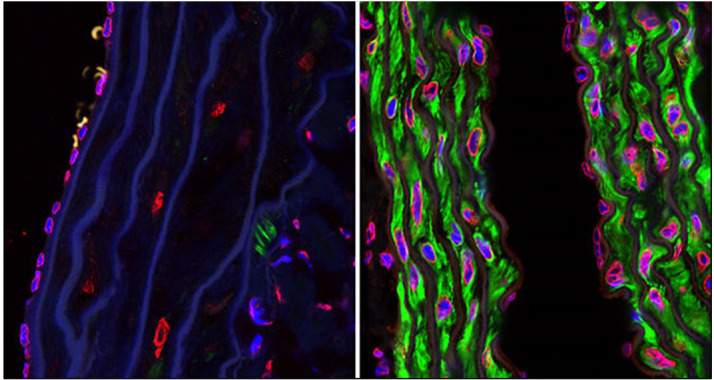
Figure 3. Confocal microscopy photographs of the descending aortas of two 15-month-old progeria mice, one untreated (left picture) and the other treated with the farnesyltransferase inhibitor drug tipifarnib (right picture). The microphotographs show prevention of the vascular smooth muscle cell loss that is otherwise rampant by this age. The staining was smooth muscle alpha-actin (green), lamins A/C (red) and DAPI (blue).

**Table:** Table1. Summary of Key Studies on HGPS and Oral Manifestations

**Study/Authors**	**Year**	**Key Findings**	**Population Studied**	**Conclusion**
Domingo et al. (5)	2009	Highlighted oral phenotypes like delayed eruption, microdontia, and caries.	Pediatric HGPS patients	Early dental issues crucial for diagnosis.
Alves et al. (12)	2014	Documented craniofacial anomalies and mandibular osteolysis.	Case study of a single patient	Importance of imaging in diagnosis.
Coutinho et al. (25)	2009	Discussed the molecular mechanisms behind oral abnormalities.	Review article	Genetic insights essential for therapies.

**Table:** Table2. Current Management Strategies for Dental Complications in HGPS

**Management Strategy**	**Approach**	**Evidence/References**
Preventive dental care	Use of fluoride therapies to prevent caries.	Domingo et al. (5), Alves et al. (12)
Multidisciplinary care	Collaboration among dentists, orthodontists, and geneticists.	Coutinho et al. (25)
Early orthodontic intervention	Management of malocclusion and dental crowding.	Alves et al. (12), Hennekam (13)
Periodontal care	Monitoring for gingivitis and periodontitis.	Domingo et al. (26)

**Table:** Table3. Genetic Studies Linking HGPS to Oral Manifestations

**Study/Authors**	**Year**	**Genetic Focus**	**Relevance to Oral Health**
De Sandre-Giovannoli et al. (1)	2003	Identified lamin A truncation as the cause of HGPS.	Explains cellular basis of oral anomalies.
Gordon et al. (22)	2016	Summarized LMNA mutation diagnostics.	Facilitates early genetic testing for dental care planning.
Cao et al. (16)	2011	Studied progerin's role in tissue-specific pathology.	Links progerin accumulation to oral tissue defects.

Hutchinson-Gilford Progeria Syndrome is a rare autosomal dominant segmental premature aging disease characterized by accelerated aging in children [1]. In 1886, Hutchinson published "Case of Congenital Absence of Hair, with Atrophic Condition of the Skin and its Appendages", making the first description of the genetic disorder now known as Hutchinson-Gilford Progeria Syndrome (HGPS). Later, Gilforde discovered this syndrome in 1904 [2]. This disorder results from a mutation in the LMNA gene, which encodes for the nuclear structural proteins lamin A and C [3]. Glycine GGC changes to glycine GGT in codon 608 of the lamin A (LMNA) gene [4]. This causes a cryptic splice donor site to activate to produce abnormal lamin A [4]. This mutation results in the production of an abnormal version of the prelamin A protein called progerin [4]. The production of progerin results in the disruption of the nuclear envelope’s integrity and results in cellular instability [4]. Children with HGPS exhibit premature aging, growth retardation, loss of subcutaneous fat, joint stiffness, and hair loss. Cardiovascular disease, especially atherosclerosis, is the primary cause of death, with affected individuals often passing away from complications in their early teens [5].

The infants appear healthy at birth. In the first few years of life, ailments and physical features often associated with the elderly start to develop.Multiple organs and tissues exhibit phenotypes that are associated with normal aging [6]. The average life expectancy of these patients is about 14.6 years.

In the context of HGPS, dental and oral aspects can present unique challenges due to underlying genetic abnormalities, and accelerated aging process. Children with HGPS present with distinct craniofacial features that affect dental development and oral health.These abnormalities include a high-pitched nasal voice, delayed dental eruption, overcrowded teeth, and micrognathia.The reduction in the capacity for cellular repair and regeneration in these patients leads to a higher susceptibility to oral diseases and complications.Comprehending these oral and dental manifestations is crucial for delivering comprehensive care to individuals with HGPS, enhancing their quality of life, and potentially prolonging their lifespan through preventive and therapeutic interventions [7].

Presently, there exists a test for HGPS. Earlier, diagnosis has to be made on physical symptoms alone. These symptoms were not present until the first or second year of the child’s life. This diagnostic test tests for an LMNA mutation and confirms a suspected diagnosis [7].

Individuals with HGPS are associated with an increased incidence of dental caries. These patients must have recall visits for reexamination of the teeth and associated hard & soft tissues of the oral cavity. Oral hygiene instructions must be advised to each patient during each dental visit [7]. The dentist should incorporate fluoride therapies into the preventive protocol for these patients. There are various fluoride therapies available, including fluoride varnishes, self-applied fluorides (toothpaste, gel, mouth rinses), and professionally applied topical fluorides (higher-strength rinses, gels, foams) [7]. It is important to emphasize that many bottled drinks of water do not contain optimal levels of fluoride [7]. Although substantial progress has been achieved in understanding the dental and oral manifestations of HGPS, numerous questions remain unanswered, presenting opportunities for further research [7].

### 1. Clinical Features

The clinical manifestations of progeria are not present at birth. Usually, affected children appear healthy at birth.Within the first two years of life, children with progeria show signs and symptoms of rapid aging that include growth failure, balding, wrinkled skin, conductive hearing loss, stiff joints, reduced mobility, scleroderma, and loss of body fat.These children often exhibit a distinctive facial appearance, characterized by a disproportionately small face relative to the head, a pinched nose, thin lips, and a small chin [7]. The skin appears wrinkled, thin, and aged [7]. as shown in Figure-1. Severe growth retardation is one of the hallmark features of progeria that result in short stature and low body weight [8]. Despite these physical challenges, their cognitive development usually remains normal, and they do not exhibit intellectual disabilities.

The skin of children with progeria undergoes profound changes that resemble accelerated aging.By the age of one or two, their skin becomes sclerotic and tight, especially over the extremities, abdomen, and buttocks.The scalp hair, eyebrows, and eyelashes may fall out leading to alopecia.

Skeletal abnormalities include delayed closure of fontanelles, osteolysis, and osteopenia, which makes the bone fragile and prone to fractures [8]. Children with progeria frequently develop joint contractures, limiting their range of motion, and they often have a stooped posture due to spinal deformities as shown in Figure-2.The affected individuals develop atherosclerosis which is the hardening and narrowing of arteries, leading to an increased risk of heart attack and strokes.These children exhibit metabolic abnormalities, including insulin resistance and abnormal lipid profiles.

Conductive hearing loss due to middle ear abnormalities is a common feature. Involvement of the gastrointestinal system causes gastrointestinal reflex and middle ear abnormalities.

### 2. Oral Manifestations

#### 2.1. Delayed Dental Development

There will be a delay in the eruption of primary and permanent teeth that leads to crowding and malocclusion. There will be a noticeable discrepancy in the size and shape of teeth [8].

#### 2.2. Abnormal Tooth Structure

Enamel hypoplasia occurs in children with progeria where the enamel becomes thin and deficient. Thus, the teeth become more susceptible to decay and wear. The dentin located beneath the enamel may be affected leading to increased tooth sensitivity and a higher risk of dental fractures [9].

#### 2.3. Periodontal Disease

The reduction in the ability of oral tissues to regenerate combined with immune system suppression predisposes children to infections. The gums become red, and swollen causing gingivitis. If treatment is not initiated, gingivitis progresses to periodontitis causing loss of teeth.

#### 2.4. Micrognathia

The mandibular jaw bone becomes underdeveloped causing smaller jaw and dental crowding. This misalignment causes difficulty in chewing, speaking, and maintaining oral hygiene [9].

#### 2.5. Oral Mucosa and Soft Tissue Changes

The oral mucosa appears thin making it susceptible to injury and ulceration. There will be reduced salivary gland function leading to xerostomia. The reduced salivary secretions increase the risk of dental caries and oral infections. Table-1 summaries all oral manifestations.

### 3. Genetic Basis of HGPS

The genetic defect behind HGPS involves a silent de novo point mutation in the LMNA gene that encodes for A-type lamins.The nuclear lamins are type V intermediate filament proteins that serve as the nuclear lamina's critical components. These critical components are located underneath the inner nuclear membrane. Nuclear lamins are categorized into A-type lamins and B-type lamins. A-type lamins include lamins A and C, two gene products that arise from alternative splicing.LMNB1 and LMNB2 encodes for B-type lamins that include lamins B1, B2, and B3. These proteins play an important role in cell division, embryonic development, and aging. Lamins serve multiple functions that include DNA replication, and transcription, and provide integrity to the chromatin and nuclear membrane [10]. B-type lamins are associated with constitutive expression in embryonic and somatic tissues [10].

In contrast, the differential expression of A-type lamins appears to be developmentally regulated, as evidence suggests that A-type lamins are expressed at higher levels in differentiated somatic cells compared to stem cells. In the initial stage, A-type lamins occur during mid-embryonic development in the head, trunk, and appendages.Other organs do not express these proteins until the early postnatal stages.The prelamin undergoes post-translational processing that leads to the production of mature lamin A protein.Prelamin A contains the "CaaX" motif at the 3’ end. A farnesyl group is added to the cysteine in the CaaX motif to produce mature lamin A.This protein becomes embedded into the cell membranes.

The "aaX" motif is removed by the action of an integral membrane protein and zinc metalloprotease called Zmpste24 or by the endoprotease known as Ras converting enzyme 1 (RCE-1).Subsequently, the isoprenyl cysteine methyltransferase (ICMT) enzyme adds a carboxymethyl group.The C-terminal end containing the farnesyl group is removed by Zmpste24 through an internal splice site in exon 11, resulting in the production of mature lamin A.The protein is not embedded into the cell membrane as the farnesyl group is no longer attached to lamin A.

Diseases that arise from the mutations in the nuclear lamins are collectively known as "laminopathies". Examples are autosomal dominant Emery-Dreifuss muscular dystrophy, restrictive dermopathy, dilated cardiomyopathy, lipodystrophy, and progeria including HGPS. In HGPS, the glycine-to-glycine mutation at position 608 results in an active cryptic splice donor, leading to abnormal splicing of the LMNA transcript in exon 11.The mutation creates a cryptic splice site while maintaining the reading frame of the transcript, resulting in the removal of 50 amino acids.

These removed amino acids include the target recognition site for Zmpste24.The silent mutation results in the permanent farnesylation of LMNA which is cleaved off in wildtype lamin A. This results in the production of a mutant lamin A isoform known as progerin.Since progerin retains the farnesyl group, the mislocalized protein accumulates in the nuclear membrane.This results in the distortion of nuclear morphology.This serves as a characteristic feature of HGPS cells. Progerin accumulates in various tissues such as the heart, skin, kidney, adipose tissue, skeletal muscle, vascular endothelial cells, and smooth muscle cells.

Several theories have been suggested to explain how the accumulation of progerin leads to the phenotypes observed in HGPS patients.Lamins A/C helps in maintaining mechanical & functional nuclear integrity.These proteins also play important roles in transcription, cell division, cellular differentiation, and DNA replication [10]. Cells isolated from HGPS patients exhibit increased levels of reactive oxygen species (ROS), resulting in increased DNA damage and premature entry into cellular senescence.

As the cells undergo multiple rounds of cell division before reaching cellular senescence, their telomeres progressively shorten. The fibroblasts that were isolated from HGPS patients undergo telomere attrition.Interestingly, progerin has been found to accumulate naturally in normal human cells and tissues. Fibroblasts isolated from older individuals had higher levels of the mutated lamin A progerin protein compared to those from healthy young individuals. Cells from these older individuals exhibited abnormalities in nuclear morphology and reduced intensity of heterochromatin markers, similar to what is observed in cells derived from HGPS patients.Normally aged cells showed increased DNA damage, as indicated by cH2AX staining.This characteristic feature, previously observed in aged mice and baboons, is also common in HGPS cells. Scaffidi and Misteli discovered that using a morpholino oligonucleotide targeting the cryptic splice site could rescue the nuclear abnormalities in older individuals. Some studies confirm progerin expression in normal individuals and different organ systems. Progerin was found to be expressed at all ages, but the mutant protein was shown to accumulate within the dermal fibroblasts and terminally differentiated keratinocytes of the skin in these individuals as summarized in Table-3.

### 4. Cardiovascular Pathology in Progeria

One of the most significant aspects of HGPS is its profound effect on the cardiovascular system. Cardiovascular pathology plays an important role in the reduced life expectancy of affected individuals, who face severe cardiovascular complications [10]. Their comprehensive analysis of cardiovascular pathology in progeria covers underlying mechanisms, clinical manifestations, and possible therapeutic strategies [10].

### 4.1. Endothelial Dysfunction

Endothelial cells, which line the interior surface of blood vessels, and are essential for vascular function, show abnormalities in progeria [11]. These include impaired cell proliferation and migration. The accumulation of progerin disrupts the nuclear envelope and alters the gene expression of endothelial cells, resulting in endothelial dysfunction [11]. This dysfunction is characterized by increased vascular permeability, decreased nitric oxide production, and impaired angiogenesis [11].

### 4.2. Atherosclerosis

Atherosclerosis, marked by the accumulation of plaques in the arterial walls, is a major cardiovascular concern in progeria [11]. Progerin accumulation in smooth muscle cells and endothelial cells disrupts normal cellular functions, facilitating the formation of atherosclerotic lesions.These lesions tend to be more severe and result in early cardiovascular events. The plaques are often laden with inflammatory cells and lipids, which worsen vascular damage.

### 4.3. Intimal Fibrosis

Intimal fibrosis is characterized by the thickening of arterial intima. The intima is the innermost layer of the arterial wall that undergoes fibrosis due to excessive deposition of extracellular matrix components [11]. The effect of progerin on smooth muscle cells and fibroblasts leads to an abnormal build-up of collagen, resulting in increased vessel and decreased arterial compliance [11].

### 4. 4. Vascular Smooth Muscle Cell Loss

Vascular smooth muscle cells help maintain the structural integrity of blood vessels. Progerin-induced toxicity leads to the premature death of vascular smooth muscle cells. The loss of these cells compromises the structural integrity of the vascular wall, leading to arteriosclerosis, which is marked by the thickening and hardening of the arteries.

### 5. Clinical Manifestations

The cardiovascular pathology in progeria leads to severe clinical manifestations, which are often present at a young age:

### 5. 1. Hypertension

Children with progeria develop hypertension due to increased arterial stiffness and decreased compliance. This condition places additional stress on the cardiovascular system [11].

### 5. 2. Stoke

Due to the atherosclerotic changes in the cerebral arteries, there is an increased risk of occurrence of cerebrovascular events such as stroke [11].

### 5. 3. Heart Failure

Myocardial ischemia and chronic pressure overload can cause left ventricular hypertrophy and heart failure. The stiffened arteries and elevated afterload hinder the heart’s ability to pump blood effectively [11].

### 5.4. Angina and Myocardial Infarction

The blood supply to the heart becomes compromised due to the narrowing of arteries resulting in myocardial infarction and angina.

### 6. Therapeutic Strategies for Treating Progeria

The detection of molecular causes of the disease initiated effective pharmacological treatment for progeria. The categories can be categorized into three main groups: (a)development of biologicals; (b) small molecule treatments; and (c)gene therapy approaches [12] as shown in Figure-3.

### 7. Development of Biologicals

Biological drugs include complex molecules such as peptides, proteins, nucleic acids, monoclonal antibodies, and vaccines [12]. The antisense oligonucleotides (ASOs) are the most advanced groups in progeria. ASOs are short sequences of nucleotides, designed to complement a specific target RNA sequence [12].

ASO binds specifically to the target RNA using Watson-Crick base pairing rules. Different chemical modifications are aimed at increasing stability and decreasing immune response [12]. One such modification involves morpholino oligonucleotides, polymers composed of standard DNA nucleobases attached to a backbone made of methylene morpholine rings connected by phosphorodiamidate linkages [12].

The first morpholino oligonucleotide complementary to the region containing the HGPS mutation in exon 11 was reported in 2005. However, the administration of this morpholino oligonucleotide did not significantly improve the cardiovascular pathology, which is a critical issue in progeria. This outcome has driven research efforts focused on optimizing the bioavailability and in vivo efficacy of ASOs [12].

To this end, Misteli’s group undertook the de novo identification of ASOs using an unbiased approach, screening a large, diverse library of molecules with various target sequences, backbone chemistries, and lengths [12]. This approach led to the identification of the ASO B143, which targets the junction of LMNA exon 12 instead of the mutated exonic splice site in exon 11[12].

B143 was conjugated with a palmitoyl acid chain to enhance cellular uptake and stability. In vivo, administration of this lipid-modified ASO, named L-B143, significantly reduced progerin mRNA levels across all tissues [12]. However, the degree of progerin protein varied between organs, indicating that progerin has a long half-life and exhibits tissue-specific turnover in vivo. L-B143 significantly extended the lifespan of a transgenic mouse model of HGPS that expresses the human LMNA gene with the classic G608G mutation [12]. This effect was observed when the compound was administered subcutaneously at doses of 17mg/kg or 50mg/kg [12].

However, some toxicity concerns were reported at the highest dose. In this case, the treated animals not only showed improved survival and an overall increase in body weight, but they also experienced a reduction in both the incidence and severity of progeria-induced hypertrophy in the media of the interstitial arteries of the heart [12].

However, the treatment did not significantly correct aortic morphology. Meanwhile, an independent study conducted by Collin’s group analyzed a series of phosphorodiamidate morpholino oligomers (PMOs) targeting the cryptic splice site in exon 11 at five nucleotide intervals [12]. The most effective PMOs were then conjugated with a peptide tag to enhance cell penetration, allowing for the compound in vivo intravenous administration to test its efficacy [12].

This approach led to the identification of the peptide-conjugated PMO SRP-2001, which achieved the most significant reduction in progerin mRNA levels in patient fibroblasts [12]. Intravenous administration of SRP-2001 (60 mg/kg twice a week) to the transgenic mouse model of HGPS, which expresses the human LMNA gene with the classic G608G mutation, resulted in approximately a 60% increase in lifespan and reversed the loss of vascular smooth muscle cells in large arteries [12].

Together, these results underscore the potential of this therapeutic approach for treating progeria [12].

### 8. Gene Therapy Approaches

Gene therapy approaches are aimed at the correction of the root of the problem, to directly repair the disease-causing mutation [12]. In 2019, two independent studies showed that using a CRISPR/Cas9-based genome-editing approach to target the LMNA exon and interfere with lamin A/progerin expression significantly improved the overall amelioration of the progeroid phenotype and extended lifespan.

A recent study has used base editors, genome editing agents, that convert targeted base pairs without making double-strand DNA breaks. The lentiviral delivery of adenine base editors (ABE) to human progeroid fibroblasts resulted in approximately 90% correction of the pathogenic allele, a reduction in RNA mis-splicing and progerin levels, and a correction of nuclear abnormalities [12][13].

In the Hutchinson-Gilford Progeria Syndrome (HGPS) mouse model, a single retro-orbital injection of adeno-associated virus 9 (AAV9) encoding ABE led to a notable correction of the pathogenic mutation, restoration of normal RNA splicing, and a reduction in progerin levels [12].

This treatment also increased the number of vascular smooth muscle cells (VSMCs) and prevented fibrosis, two key indicators of cardiovascular damage in progeria. Remarkably, a single injection of AAV9 expressing ABE on postnatal day 14 improved vitality and significantly extended the median lifespan of the mice from 215 to 510 days, representing the most substantial lifespan increase observed with any experimental therapy to date. Despite these impressive and promising results, which underscore the potential of ABE base editors as a treatment for progeria, several limitations need to be addressed, such as the risk of exacerbated immune responses or the induction of liver tumors, before this therapy can be applied to patients [12][14].

### 9. Rapamycin and Everolimus

These drugs inhibit the mTOR pathway which plays a role in metabolism and cell growth [17]. Rapamycin enhances progerin clearance by promoting autophagy, a cellular process responsible for degrading damaged proteins [17]. Preclinical studies have identified that rapamycin treatment can improve nuclear morphology and extend the lifespan of progeroid mice [17].

### 10. Statins and Bisphosphonates

Statins, typically used to lower cholesterol levels, and bisphosphonates, which are used to treat osteoporosis, have been explored for their potential benefits in progeria [19]. These drugs work by reducing the prenylation of progerin. Preclinical studies have demonstrated modest improvements, but their effectiveness in human patients is still being investigated [19].

Sulforaphane is an antioxidant that has been used to enhance progerin clearance by autophagy. It is obtained from cruciferous vegetables. Both Sulforaphane and Lonafarnib have been used in the treatment of HGPS [21].

### 11. Supportive Care and Symptomatic Management

A regular diet along with persistent meals is advised. Deciduous tooth extractions can be performed after permanent tooth eruption to avoid dental crowding [22]. Hip dislocation can be managed through body bracing and physical therapy. Hydrotherapy, strengthening exercises, active stretching, routine physical, and occupational therapy are recommended [22].

Maintaining optimal hydration is essential along with physical activity to minimize the risk of stroke [22]. Modified transcatheter aortic valve replacement can be used to treat critical aortic stenosis that can result in an increased life span [22].

Cardiovascular complications are the primary cause of mortality in individuals with progeria. Continuous monitoring of heart health is essential, and treatments may include medications to manage conditions such as hypertension and hyperlipidemia [22]. In severe cases, surgical procedures like coronary artery bypass grafting may be required to address significant cardiovascular issues as discussed in Table-2 [22].

### 12. Physical and Occupational Therapy

Physical and occupational therapy are critical for maintaining mobility and enhancing quality of life due to joint stiffness and mobility changes. Customized exercise programs can help preserve muscle strength, flexibility, and overall physical function [23].

### 13. Nutritional Support

Children with progeria experience difficulties with nutrition due to growth delays and other metabolic concerns. High-calorie diets and supplements may be necessary to ensure proper growth and development [23].

### 14. Psychosocial Support

Coping with a rare and life-limiting condition like progeria can be emotionally challenging for both patients and their families. Psychosocial support, such as counseling and support groups, can address the emotional and psychological aspects of the disease [23].

## Conclusion

The dental and oral implications of Hutchinson-Gilford Progeria Syndrome highlight the necessity for comprehensive and specialized care for these patients. Despite the significant challenges the condition presents, early detection, preventive strategies, and a multidisciplinary approach can effectively address dental and oral complications, ultimately enhancing the quality of life for those affected. Ongoing research and clinical advancements are promising, potentially leading to more effective management and treatment options, providing hope for better outcomes for individuals with HGPS [24].

## Conflict of Interest

None.
